# Fibrosarcoma of Mandible: A Case Report

**DOI:** 10.1155/2011/536086

**Published:** 2011-07-07

**Authors:** Monal B. Yuwanati, J. V. Tupkari

**Affiliations:** Department of Oral Pathology and Microbiology, Government Dental College and Hospital, Mumbai, India

## Abstract

Fibrosarcoma is a malignant mesenchymal neoplasm of fibroblasts that rarely affects the oral cavity and can cause local recurrences or metastasis. In this paper, a case of fibrosarcoma in the posterior area of mandible in a 44-year-old woman is described. Clinical examination revealed a growth on right mandibular third molar region extending on the buccal and the lingual side. There was history of extraction of posterior teeth. Radiologically, there was a diffuse bone loss. Microscopically, the tumor showed an intense proliferation of fibroblasts of variable size and shape. These cells were arranged in parallel bands and partly crossing each other. The cells exhibited increased mitotic activity and nuclear pleomorphism. Immunohistochemically the cells showed immunoreactivity only for vimentin while they exhibited negativity towards S-100 protein, cytokeratin cocktail, HMB-45, desmin, smooth muscle actin, and epithelial membrane antigen (EMA). Based on findings the final diagnosis of fibrosarcoma was made.

## 1. Introduction

Soft tissue sarcomas are rare in the oral and maxillofacial region and account for less than 1% of the cancers. At one time, fibrosarcoma was the most common soft tissue sarcoma. With the introduction of electron microscopy and immunohistochemistry, it became evident that many previously diagnosed fibrosarcomas were other spindle cell malignant lesions. Fibrosarcoma is defined as a malignant spindle cell tumor showing a herringbone or interlacing fascicular pattern without expression of other connective tissue cell markers. Fibrosarcoma is a malignant neoplasm of the fibroblastic origin. It has been reported in association with several conditions, such as Paget's disease and fibrous dysplasia and postradiotherapy. Fibrosarcoma of head and neck area represents 5% of all malignant intraosseous tumors. It can occur in any location but mainly affects long bone particularly, and its occurrence in craniofacial region is about 15%, mandible being the most common site. Although fibrosarcoma has been reported in all groups, it is most commonly seen in the 3rd and 6th decades of life [1]. The patient classically presents with pain, with or without swelling. In oral cavity, loosening of teeth may be apparent. This paper describes a case of fibrosarcoma of the mandible.

## 2. Case Report

**Figure 1:**
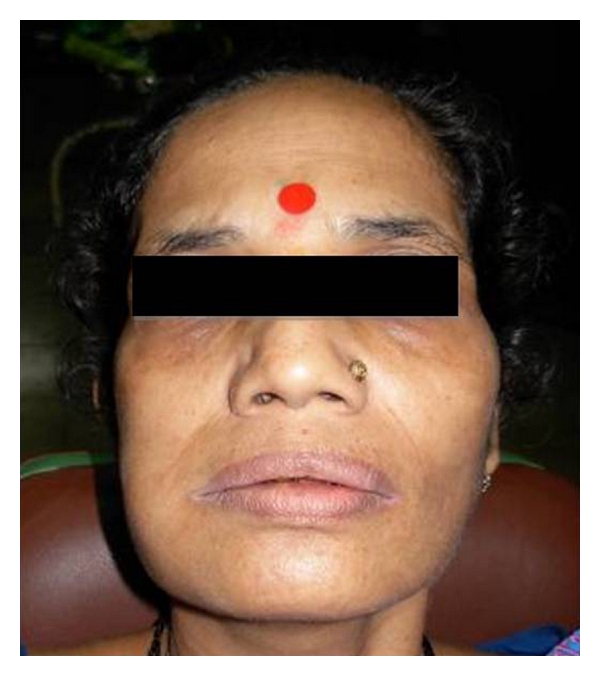
Extraoral examination revealed diffuse enlargement of lower right side of face over the angle and body of mandible.

**Figure 2:**
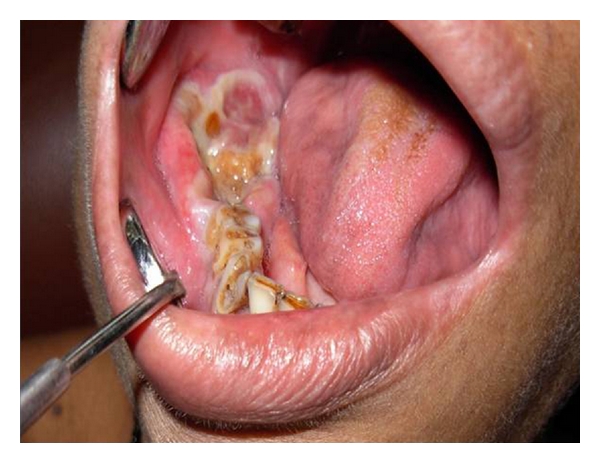
Ulceroproliferative growth seen on lower right posterior third molar area.

A 44-year-old female was referred to the Department of oral Pathology, Government dental College and Hospital, Mumbai. She complained of a painful swelling in the right side of mouth (Figure 1). The patient also claimed that her right-sided wisdom tooth was extracted about 15 days back due to mobility. Since then she has been experiencing pain in the posterior area of the mandible associated with swelling. Paresthesia was evident in the right lower lip. The patient's medical history was noncontributory. On intraoral examination, large ulceroproliferative mass was observed in the right-side posterior mandible (nos. 46, 47, and 48). The lesion was soft to firm and measured about 4 cm × 3 cm (Figure 2).

**Figure 3:**
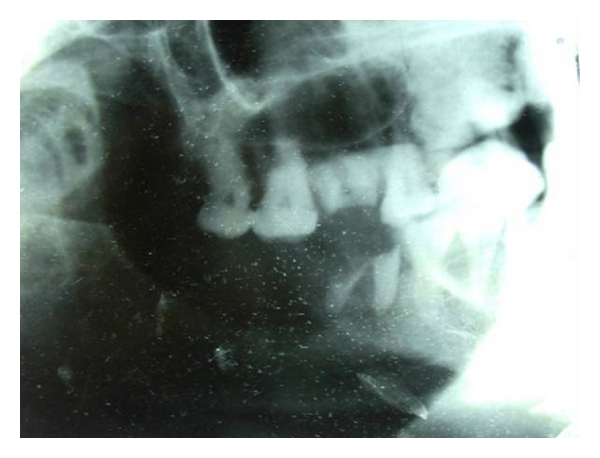
Lateral oblique—osteolytic lesion in nos. 46, 47, 48 regions. S/o destructive bony lesion.

**Figure 4:**
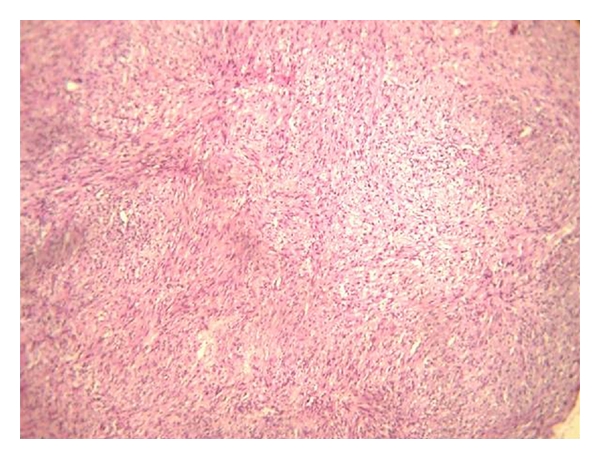
Photomicrograph showing high cellularity, intense proliferation of spindle-shaped cells and variable deposition of collagen (10x view).

**Figure 5:**
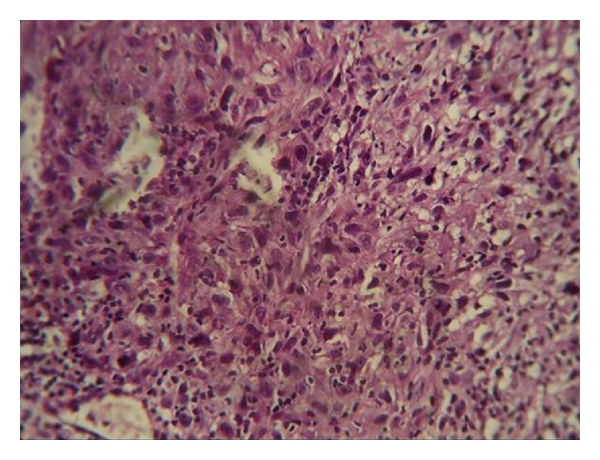
Spindle shaped cell showing nuclear pleomorphism (40x).

**Figure 6:**
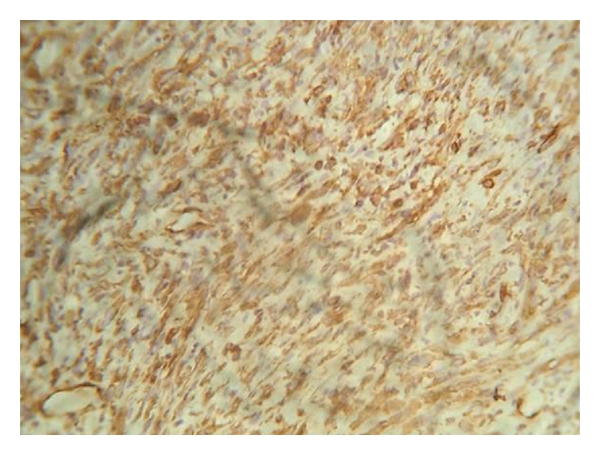
Neoplastic cells displaying intense immunoreactivity for vimentin (40x).

 Radiological examinations with lateral oblique radiograph (Figure 3) showed osteolytic areas with ill-defined borders located in the right mandibular angle and ramus. An incisional biopsy was performed, and on microscopic examination fascicles of spindle-shaped cell with areas of collagen fibers were observed (Figure 4). The cells exhibited hyperchromatic nuclei and increased nuclear cytoplasmic ratio (Figure 5). The microscopic aspect of the investigations led on to the diagnosis of fibrosarcoma. Further, immunohistochemistry analysis showed a positive reaction for vimentin (Figure 6), but not for S-100 protein, cytokeratin cocktail, HMB-45, Desmin, smooth muscle actin, and epithelial membrane antigen (EMA). Upon confirmation of the presence of fibrosarcoma, the patient was referred to higher centres for further evaluation and treatment. The patient did not report for followup.

## 3. Discussion

Fibrosarcoma is malignant mesenchymal tumor of fibroblast. Although it can occur in any location, the bone extremities are the most commonly affected site. Primary fibrosarcomas are rare in mandible which is common site in jaws (Table 1). It is often difficult to determine whether the lesion primarily developed in the soft tissue or intraosseously in the head and neck. Intraosseous fibrosarcomas may develop endosteally or possibly periosteally, the latter affecting bone by spread from adjacent soft tissue to present a clinical and radiographic appearance of primary bone lesion [2]. However, others accept that the fibrosarcoma of bone as a distinctive lesion can arise in preexisting benign lesions such as ameloblastic fibroma, chronic osteomyelitis, Paget's disease, fibrous dysplasia, and giant-cell tumor of bone [3]. 

A fibrosarcoma arising in the region of a dental extraction has been reported [4] in past. Usually primary intraosseous fibrosarcoma is asymptomatic and appears as slow growing mass which was not in our case. It was associated with mobility of adjacent teeth and ulceration of overlying mucosa. Pain and paresthesia are usually late symptoms indicating nerve involvement.

Radiographically, fibrosarcoma often appears as a purely osteolytic lesion without calcification and with poorly defined, irregular margins if it has arouse intraosseously. There is usually destruction of the cortical plates without expansion [5], and the lesion may be misdiagnosed as an odontogenic abscess or cyst. The roots of adjacent teeth may or may not show resorption [5].

 The classical fibrosarcoma has been characterized microscopically by uniform spindle cells distributed in interlacing fascicles with herring bone growth pattern. In the present case, the lesion classically composed of pleomorphic spindle-shaped cells arranged as bands or interweaving fascicles with variable collagen and collagen reticulin production and degree of anaplasia. Mitosis may be sparse or plentiful. The integral vascularity of fibrosarcoma with lack of proper endothelial lining has been emphasized as a differential point.

 In differential diagnosis, reactive fibromatosis, fibroblastic osteogenic sarcoma, pseudosarcomatous fasciitis, and cellular alveolar sarcoma must be excluded. The positive immunostaining for vimentin, together with negativity for muscular immunomarkers, will help establishing the diagnosis of the fibrosarcoma.

 The treatment of choice is surgical resection with a wide margin. The need for adjuvant radiotherapy and/or chemotherapy is still unclear and is normally indicated in high-grade tumors because these tumors may present subclinical or microscopic metastases at the time of diagnosis. In addition, prophylactic neck dissection is controversial. In Our case, details of treatment and follow up information of the patient were not available as patient did not undergo the treatment of surgical resection, chemotherapy, and radiotherapy. The overall survival rate at 10 years may vary from 21.8% to 83%, and clinical stage, histological grade of malignancy, and local recurrences are the most important prognostic factors.

 In recent years, the fibrosarcoma of head and neck area has been increasingly reported but still there are paucity of reports of fibrosarcoma of head and neck region. Hence, it should be included in differential diagnosis, especially in cases of rapidly growing lesions in the mouth.

## Figures and Tables

**Table 1:** Cases of primary fibrosarcoma of mandible reported in the literature.

Author	Year	No.	Site	Treatment	Recurrence	Followup
Gosau et al.	2008	01	Mandible (1)	surgery	No	3 yrs
Orhan et al.	2007	01	Mandible (1)	Surgery + RT + CT	NA	NA
Borges Soares et al.	2006	01	Mandible (1)	Radical surgery	No	1 yrs 9 months
Pereira et al.	2005	01	Mandible	Radical surgery	No	36 months
Yamaguchi et al.	2003	03	Mandible (3)	Surgery	No	9 yrs
L. Lo Muzio et al.	1998	01	Mandible	Radical surgery	No	4 yrs
Lillenget al.	1997	01	Mandible	Surgery + RT	Local + lung	21 yrs
Sadoff and Rubin et al.	1990	01	Mandible	Surgery	Local	NA
Moloy et al.	1989	01	Mandible	Surgery	Local+ regional	6 months
Taconisand van Russel et al.	1886	14	Mandible (10)	Surgery + RT	Local + Lung	NA
Handlers et al.	1985	01	Mandible	Surgery + RT + CT	Local	15 months
Zachariades and Papanicolau et al.	1985	01	NA	NA	NA	NA
Slootweg and Mulles et al.	1984	07	Mandible (5)	NA	NA	NA
Lam et al.	1979	03	NA	NA	NA	NA
Ferulito et al.	1979	01	Mandible	NA	NA	NA
Looser and Kuehn et al.	1976	04	Mandible (4)	Surgery	NA	NA
Jeffreeand price et al.	1976	07	Mandible (4)	NA	NA	NA
Huvos and Higinbotham et al.	1975	12	Mandible (10)	NA	NA	NA
Haidar et al.	1975	01	Mandible	NA	NA	NA
Van Blarcom et al.	1971	13	Mandible (13)	NA	NA	NA
Jochimsen and Grage et al.	1971	01	Mandible	NA	NA	NA
Monaly Uwanati and J.V.Tupkari et al.	2011	01	Mandible	NA	NA	NA
